# CT‐less electron radiotherapy simulation and planning with a consumer 3D camera

**DOI:** 10.1002/acm2.13283

**Published:** 2021-05-27

**Authors:** Lawrie Skinner, Rick Knopp, Yi‐Chun Wang, Piotr Dubrowski, Karl K. Bush, Alyssa Limmer, Nicholas Trakul, Lynn Million, Carol M. Marquez, Amy S. Yu

**Affiliations:** ^1^ Stanford Radiation oncology Palo Alto CA USA

**Keywords:** 3D camera, CT simulation, electron beam, radiotherapy, surface imaging

## Abstract

**Purpose:**

Electron radiation therapy dose distributions are affected by irregular body surface contours. This study investigates the feasibility of three‐dimensional (3D) cameras to substitute for the treatment planning computerized tomography (CT) scan by capturing the body surfaces to be treated for accurate electron beam dosimetry.

**Methods:**

Dosimetry was compared for six electron beam treatments to the nose, toe, eye, and scalp using full CT scan, CT scan with Hounsfield Unit (HU) overridden to water (mimic 3D camera cases), and flat‐phantom techniques. Radiation dose was prescribed to a depth on the central axis per physician’s order, and the monitor units (MUs) were calculated. The 3D camera spatial accuracy was evaluated by comparing the 3D surface of a head phantom captured by a 3D camera and that generated with the CT scan in the treatment planning system. A clinical case is presented, and MUs were calculated using the 3D camera body contour with HU overridden to water.

**Results:**

Across six cases the average change in MUs between the full CT and the 3Dwater (CT scan with HU overridden to water) calculations was 1.3% with a standard deviation of 1.0%. The corresponding hotspots had a mean difference of 0.4% and a standard deviation of 1.9%. The 3D camera captured surface of a head phantom was found to have a 0.59 mm standard deviation from the surface derived from the CT scan. In‐vivo dose measurements (213 ± 8 cGy) agreed with the 3D‐camera planned dose of 209 ± 6 cGy, compared to 192 ± 6 cGy for the flat‐phantom calculation (same MUs).

**Conclusions:**

Electron beam dosimetry is affected by irregular body surfaces. 3D cameras can capture irregular body contours which allow accurate dosimetry of electron beam treatment as an alternative to costly CT scans with no extra exposure to radiation. Tools and workflow for clinical implementation are provided.

## 1 INTRODUCTION

Electron beam dosimetry depends on both the patient surface contour and tissue heterogeneity (density and composition variations). Although the scattering of electrons creating dose variations is well understood,^1^, ^2^ clinical electron treatments are often calculated without any volumetric imaging by assuming a flat patient surface, and a homogenous water equivalent tissue. These treatments do not account for the shape of the treated surface, which can create local dose variations in excess of ±20% for surface shapes similar to those of the nose, ear, or lips.^3^ The presence of air cavities and bone in the treatment field may also perturb the dose. To obtain more accurate dosimetry, a treatment planning computed tomography (CT) scan can be performed, and dose calculated using a three‐dimensional (3D) dose calculation algorithm. However, many electron treatments do not require visualization of internal anatomy as the treatments are directed at superficial sites where the tissue is relatively homogenous. These scans can be costly, expose patients to unnecessary ionizing radiation, and add extra time and resources for radiation therapy staff.

Techniques to improve electron beam dosimetry for irregular surfaces are limited when CT scans are not indicated. Novel approaches are needed which led us to investigate the feasibility of 3D cameras to capture body surface contours to improve electron dose calculations. Although 3D cameras have been available for over a decade, recent software developments, and reduced hardware costs have increased their accessibility. 3D cameras with sub‐mm spatial resolution are used in radiation oncology for patient motion management,^4^, ^5^ extending the CT body surface,^6^ collision avoidance,^7^ facial recognition,^8^ and electron beam aperture definition.^9^ Water‐equivalent bolus is often needed for electron treatments to increase skin dose and/or limit the dose beyond the target. In such cases, a 3D camera captured surface can be used to produce a 3D printed customized bolus and treatment aperture without the need for CT scans.^10^, ^11^

This report studies the feasibility and accuracy of spatial resolution for 3D cameras to capture irregular body contours for electron treatments including the dosimetric differences among plans based on flat phantoms, full CT scans, and CT scans overridden Hounsfield Unit (HU) to water (homogenous). Camera spatial accuracy is tested through comparison of the captured surfaces to CT scans. A clinical case and workflow are presented.

## 2 MATERIALS AND METHODS

This is a quality improvement project which is exempt from our Institutional Review Boards (IRB) and Scientific Review Committee (SRC). Due care should be taken for any workflows or software that exports patient data outside of the hospital IT infrastructure.

### 2.1 CT density override calculations

To investigate the dose differences due to the patient‐specific body contour, six clinical plans with surface topology that varies in height by at least 1cm within the treatment field were calculated in three levels: First with full CT scans (CT) accounting for both surface‐shape and tissue heterogeneity, second with the body volume overridden to water (3Dwater), accounting for surface‐shape only, and third calculated on a flat water‐equivalent phantom (flat) (see Table 1 for further details of the treatment plans).

**Table 1:** Plan parameters for each plan. Patients 1‐6 were used to validate the accuracy of a homogenous calculation compared to a full CT scan. Patient 7 had only a 3D camera‐based plan.

Patient	Dose (cGy/fx)	Treatment site	Prescribed depth^a^ (cm)	Energy (MeV)	CT scan
1	200	Nose	4.1	12	Yes
2	333	Nose	2.5	9	Yes
3	200	Eye	3.6	12/16^b^	Yes
4	800	Toe	2.7	12	Yes
5	200	Maxilla	2.8	9	Yes
6	200	Scalp	1.9	6/9^b^	Yes
7	200	Cheek	2.3	9	No

^a^ The prescribed depth was determined by the physician based on the clinical judgment.

^b^ Mixed energy of two fields using the same field aperture.

All the plans were prescribed to a point on the central axis that receives 100% of the prescription dose. To minimize uncertainty, the electron Monte Carlo (eMC) algorithm with Varian Eclipse Treatment Planning System (TPS, v15.6, Varian medical systems Palo Alto CA, USA), was calculated to 1% uncertainty with 50% dose being the cutoff for uncertainty evaluation. Medium strength smoothing was used. These settings have been found to be in good agreement (better than 3% or 3mm) with MC calculations including the central axis PDD curves.^12^, ^13^ The prescribed depth was determined by the physician based on the clinical judgment. The flat‐phantom and 3Dwater plans were also prescribed to the same physical depth on central axis as the full CT plans. All plans were calculated using eMC with a 2 mm calculation grid size and a 1% uncertainty limit. The flat phantom used a 30x30x30 cm^3^ water equivalent cube en‐face to the incident beam at the same source‐surface distance (SSD) as the full CT plan. Plans with a mixture of 100 cm and 105 cm SSDs were used. Plans were calculated on either C‐series or TrueBeam Varian linacs, using one of the 6, 9, 12, and 16 MeV electron energies. Both linac types used their respective representative beam data for Eclipse.

### 2.2 Three‐dimensional camera accuracy

**Fig. 1:**
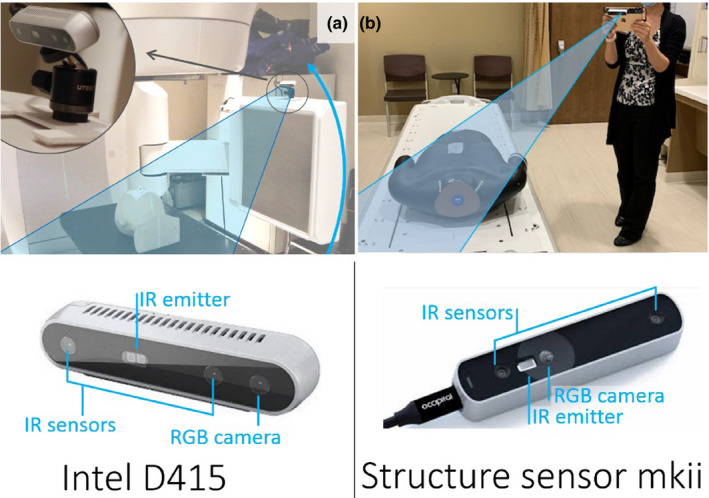
(a) An Intel D415 camera is mounted on the On‐Board Imaging system. The camera is mounted on a ball head camera mount which is screwed into a 3D printed plate that clips on the kV detector cover (the inserted figure). This setup provides a rapid and unobtrusive way to scan the full patient surface in the treatment position using the gantry rotation (blue arrow). (b) Occipital Mark ii Structure sensor and the accompanying iPad mini hand‐held setup. It is easy to use while patient is in the preparation room or exam room.

Two cameras were tested and used to obtain 3D surface scans, namely an intel D415 stereo depth camera (Intel, Santa Clara, CA, USA) connected to a laptop mounted on the side of the linac gantry [Fig. 1(a)], and a Mark ii Structure sensor (Occipital, Boulder CO, USA) connected to an iPad mini [Fig. 1(b)]. A limitation of 3D surface acquisitions is that only the visible surface may be captured. This means the patient’s body that is in contact with the couch may not be acquired. A CT scan of a head phantom (CIRS, VA, USA) was acquired with a 1.25 mm slice thickness. The CT image of the head phantom was imported to the TPS and the body contour was automatically generated by the contouring module. 3D camera scans were obtained as.obj or.stl polygon mesh files, which were converted into CT structures using 3D Slicer (https://www.slicer.org/). The registration technique used was similar to that described previously.^14^ Both the 3D camera and CT scans were imported into the TPS and an initial rough manual registration was performed. For analysis a fine registration with a least squares method was used.^6^, ^15^

To ensure a fair comparison, both the 3D camera and CT scanned structures were exported from the TPS and converted into.stl files. That is the same file formats and algorithms were used for both the CT and 3D camera surfaces. Note that.stl files consist of a list of triangle coordinates that constitute a 3D surface mesh. This mesh is a model constructed based on the x,y and depth information which the camera receives from each pixel. As the camera is moved the 3D surface mesh is refined. This process requires the patient being modeled to remain still during acquisition. The detail and accuracy of the surface mesh are dependent on the software, hardware, angles of view, and reflectivity of the surfaces captured. For the OBI mounted D415 camera we used a frame rate of 30 frames per second, and camera motion of approximately the full gantry rotation speed of the Varian 21Ex (4 degrees/s). The 3D camera and CT scanned body surfaces were then compared using CloudCompare (version 2.10, https://www.danielgm.net/cc/). As we are not concerned with the absolute position of the body surface in space, only the shape and size of the body surface reproduced by the 3D camera have been evaluated. The two surfaces were registered together using a least square algorithm. This method assumes that the body structure derived from the CT scanned surface is a “true” reference. Once registered, a histogram of distances between the 3D‐camera surface and CT‐surface was generated. For treatments, as the 3D cameras also capture a color surface map, a pen mark may be used to indicate the beam center and field edge borders which can be identified on the 3D colormap.

### 2.3 Clinical workflows and example cases

To further test the 3D camera in a clinical setting, a volunteer patient, a 65‐year‐old male with basal cell carcinoma on the skin of the nose, with an existing CT scan was scanned with the 3D camera at simulation. The monitor units (MUs) were calculated on both CT image and the 3D‐camera captured body. Although full quantitative analysis was not performed, this case, which also has a CT scan of the same anatomy, provided confidence that the 3D surface could be captured on a real patient, as well as on a plastic phantom, which was quantitatively evaluated for the shape and size of the body surface relative to the CT scan.

**Fig. 2:**
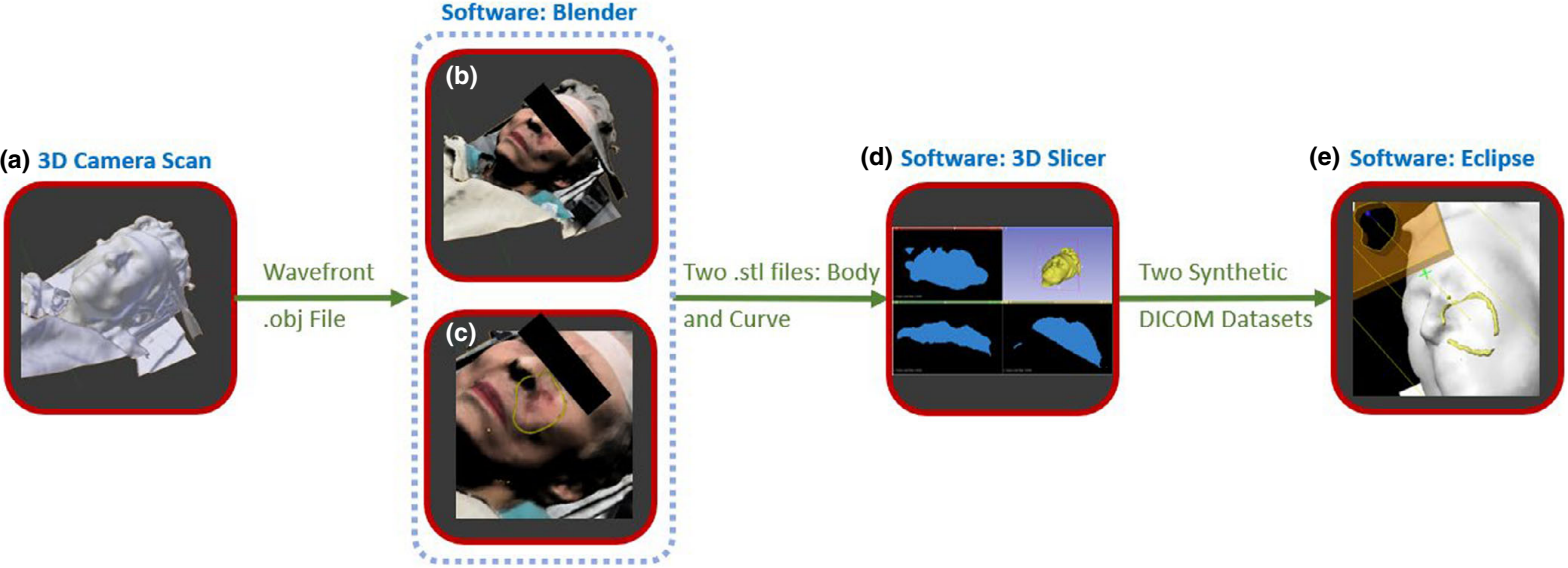
A flowchart of how to import colormap information into TPS (a) Acquire patient’s 3D surface and color information. (b) The 3D model and color map information are imported. (c) A 3D curve added manually to patient surface model tracing physician’s markings. (d) .stl meshes converted to two synthetic sliced DICOM datasets (e) Synthetic datasets are used to create structures for field aperture creation and dose calculation.

Once the accuracy of both camera and dosimetry was evaluated, the full clinical workflow was developed (see Figs. 2 and 6). For the following second clinical case, only the 3D camera body surface was captured, and the skin dose was measured. The patient was an 89‐year‐old female with a primary cutaneous CD30 positive T‐cell lesion of the left cheek. A total dose of 24 Gy was prescribed to be delivered in 12 fractions (2 Gy/Fx) using 9 MeV electron beam, and a 1 cm bolus such that 100% of the prescription dose was received at a 1.3 cm depth in the patient. The beam aperture was clinically drawn on the patient’s skin by the physician and traced by the radiation therapist onto an acrylic sheet placed in the electron applicator. The field length and width were measured to be 4.2 × 3.0 cm^2^ at 100 cm SSD. The CAX was marked on the patient’s skin when the physician was marking the treatment area. By using the 3D camera in color mode both a 3D mesh and the corresponding color map can be saved and thus the physician’s CAX mark and beam aperture outline information are captured in 3D.

Importing this information into Blender 3D modelling software (The Blender Foundation, Amsterdam) we were able to reconstruct the full‐colored 3D model of the patient, which clearly showed physician’s demarcations. A small 3D‐Freehand object was added along the surface of the 3D patient mesh carefully retracing the physician’s treatment area and CAX marks. This new object and original patient 3D surface were exported as two separate.stl files and individually converted into CT sets using 3D Slicer (www.slicer.org). The conversion takes the 3D mesh volume of each.stl file and converts it into CT slices, where the CT number inside and outside each mesh volume is given different values. These two resultant DICOM datasets (representing patient’s 3D mesh and physician’s demarcations) were finally imported into Eclipse TPS and using thresholding tools, contours of the body and physician’s marks were easily created which allowed for bolus creation, electron cutout design, and dose calculation. A flow chart is showed in Fig. 2.

Once in Eclipse the volume of the body contour was assigned as the properties of water. The planned SSD was 105 cm. Delivered dose was measured on the patient surface with an optically stimulated luminescent dosimeter (OSLD, Landauer, Glenwood, IL, USA). OSLD measurements were performed on the patient’s skin around the center of the aperture under a 1‐cm bolus and placed by the physician. A therapist took a picture of the placement of the OSLDs as the reference to look for the dose in the TPS for comparison.

## 3 RESULTS

### 3.1 Plan calculation comparisons

**Fig. 3:**
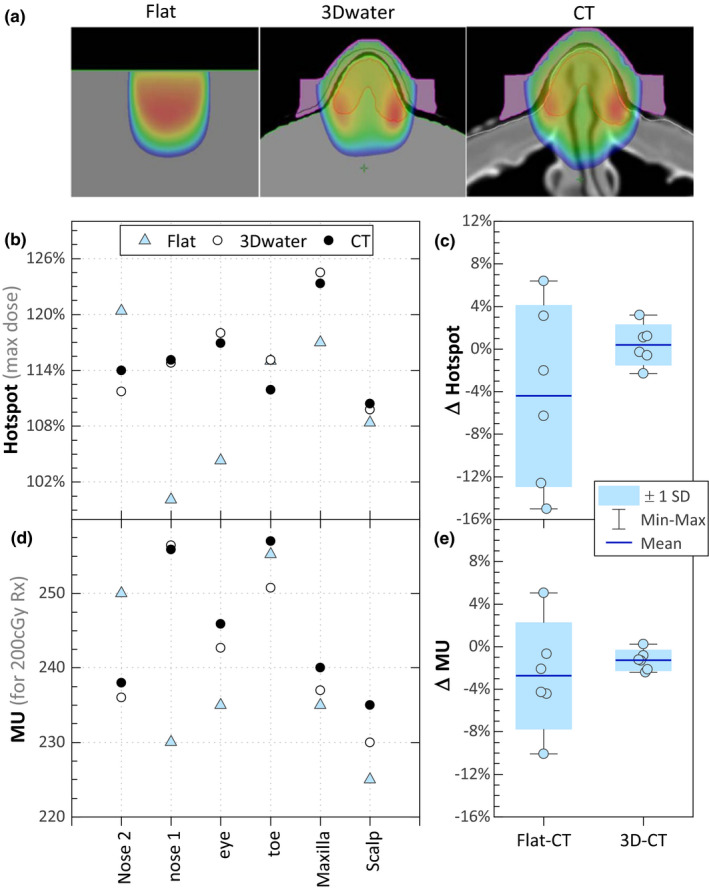
Three levels of calculation were compared. (a) Color wash ranges from 80% to Dmax, for a flat‐water phantom (Flat), water‐filled body contour (3Dwater), and full CT scan (CT). Note: a 1 mm margin between the body and 3D printed bolus is used to increase robustness of the fit. (b) The calculated hotspots for the three levels of calculation. (c) The differences in hotspot for flat phantom and 3Dwater calculation compared to the full CT scan. (d) The calculated monitor units for the three levels of calculation. (e) MU differences for the flat phantom and 3Dwater calculations compared to the full CT (plans were all prescribed to a depth on central axis per physician’s order).

Six clinical cases were investigated and compared for the three levels of dose calculation, water‐filled body contour (3Dwater), flat‐phantom (flat), and full CT (CT). The mean MU difference between the full CT and the 3Dwater calculations was 1.3% ± 1.0% (mean ± SD). Plans were normalized to the same prescribed depths [Fig. 3(e)]. The hotspots between the full CT and 3Dwater calculations had a mean difference of 0.4% and standard deviation of 1.9% [Fig. 3(C)]. The flat‐phantom calculations, in contrast, generally underestimated the hotspot value compared to the CT scan, with a mean difference of 4.4% and a standard deviation of 8.5%.

### 3.2 Accuracy of the 3D camera

**Fig. 4:**
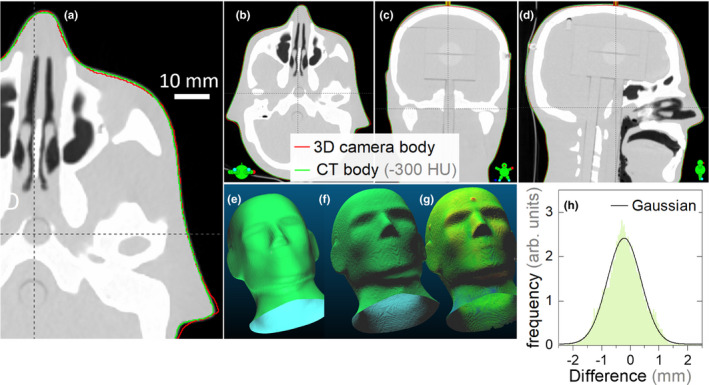
The hand‐held camera spatial accuracy test using a head phantom. (a–d) The CT scan of the head phantom with the 3D camera body contour (Red line) and CT body contour (green line). (e and f) The 3D camera captured contours before processing through Eclipse and after exporting from Eclipse. (g) The CT body contour with color indicates distance to the 3D camera captured contour. (h) The histogram of separations between the points in the CT and 3D‐camera derived body contours with a standard deviation of 0.59 mm.

Figures 4(a)–4(d) show the body contour generated by the TPS (with a −300HU threshold) from the CT data, and the body contour captured by the 3D camera (structure sensor II camera). The 3D camera contours, before processing through Eclipse and after exporting from Eclipse are showed in Figs. 4(e) and 4(f). The overlap of two groups of point clouds obtained from the CT scan and 3D camera is showed in Fig. 4(g). A histogram of the differences between the 3D camera and CT‐scanned body contours, after least squares registration in CloudCompare, is showed in Fig. 4(h). From this, the 3D camera and CT‐scanned surfaces were found to follow a simple Gaussian distribution with a standard deviation of 0.59 mm, that is, 95% of the points were within 1.2 mm in the two body surfaces.

### 3.3 Clinical cases and workflow

**Fig. 5:**
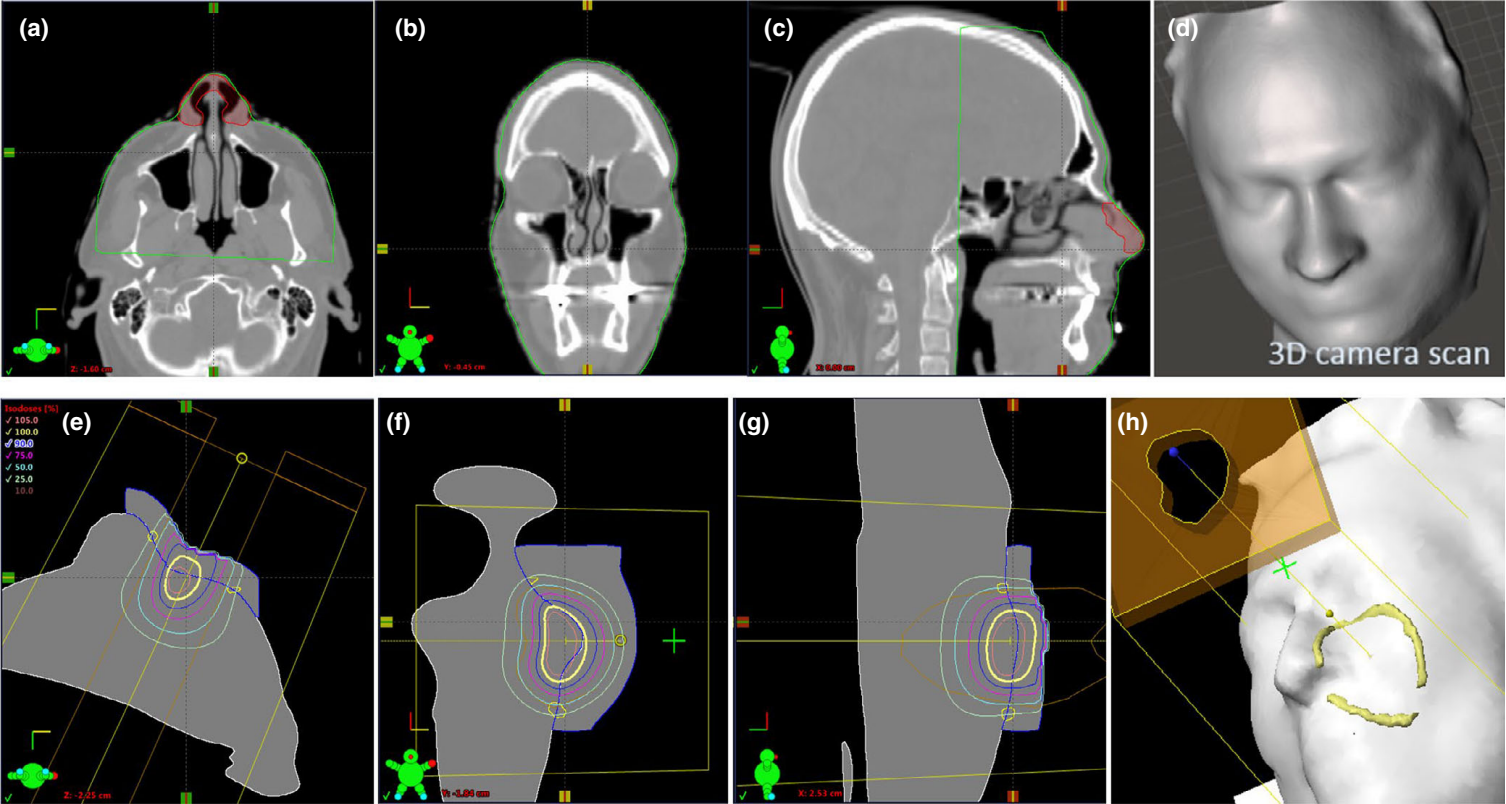
(a–d) 3D camera captured contours (green line) with its full CT scan demonstrate the 3D camera accuracy on a human patent. (e–g) No CT scan was performed, only a 3D camera scan. The bolus was created using the 3D camera captured contours. (h) The yellow outline is derived from the colormap in the 3D camera scan which was used to segment the field borders that were defined with ink marks on the patient’s face by the physician.

For the first clinical case, the patient had both CT scan and 3D camera captured surface. A plan was calculated for a clinical treatment of a nose. Figures 5(a)–5(d) show the 3D camera captured surface (green line) and the corresponding CT scan. Although the 3D camera scan was made without a mask and the CT scan was performed with a mask, qualitative assessment confirms that the camera accuracy is very close to that obtained on the phantom. The dose comparison is shown in Fig. 3 (case of nose 1).

For the second clinical case, only the 3D camera was used to capture the body surface and the skin dose was measured with OSLDs. To obtain a registration point for clinical treatments, the color of the 3D surface map was used to identify pen marks that can be used to define the beam center and treatment field edge analogous to the fiducial markers and wires used in CT scans. A plan was calculated for a clinical treatment of the left cheek. Figures 5(e)–5(g) show the 3D camera derived plan with 1 cm bolus and the calculated dose distribution. The colormap in the 3D camera scan was used to segment the field borders that were defined with ink marks on the patient’s face by the physician which was used to generate the electron aperture [Fig. 5(h)]. OSLD measurements were performed on the patient’s skin around the center of the aperture under the 1 cm bolus. As might be expected from electrons scattering into the concave surface shape,^1^, ^2^, ^3^ both the planned and measured doses, 209 ± 6 cGy and 213 ± 8 cGy (Mean±SD, Table 2), respectively, were higher than the flat phantom calculation which gave 192 ± 6 cGy for the same SSD, aperture, and MUs, that is, the measured and 3Dwater calculated doses are high because the MUs were calculated according to the prescription assuming a flat surface and a water equivalent medium. The surface shape is the only significant difference between the two calculations. Hotspots of this, 5‐10% magnitude, are expected for curved surfaces.^1^, ^2^, ^3^, ^12^, ^13^

**Table 2:** *In‐vivo* dosimetry along with planned dose (3Dwater calculation) at those locations (calculation shown in Figs. 5(e) and 5(h)). The error in the planned dose is from the range of values around the location of the OSLDs, i.e. the estimated dose error from the nanodot location uncertainty. The error in the nanodot values is the 5% uncertainty stated by manufacturer.

Location	Plan (cGy) **(mean ± SD)**	Meas (cGy) **(mean ± SD)**	Diff (%)
1	214 ± 4	230 ± 12	+7%
2	204 ± 12	196 ± 10	‐4%

## 4 DISCUSSION

Electron radiation therapy dose distributions are affected by irregular body surface contours, for example, nose, toe, eye, and scalp. A CT scan can provide greater accuracy calculating electron beam dosimetry for these sites; however, CT scan can be costly, expose patients to extra ionizing radiation, and add extra time and resources for radiation therapy staff. This study investigates the feasibility of 3D cameras to substitute for CT scan by capturing the body surfaces to be treated for accurate electron beam dosimetry. The flat‐phantom calculations, in contrast, generally underestimated the hotspot value compared to the CT scan, that is. the dose differences relative to the CT calculated plans were larger for the flat‐phantom calculations as compared to the 3Dwater calculations (a mean difference of 0.4% and standard deviation of 1.9%). The CT‐based calculations serve as the ground truth among the three methods because they have both density and surface contour information. Assuming that the CT‐based calculations are the most accurate among the three methods, the tighter spread of differences shown in Fig. 3(E) for the 3Dwater calculations indicates that it is more accurate than flat‐phantom calculations. Given these improvements over flat‐phantom calculations, 3D camera spatial accuracy was then investigated as a means of implementing the CT‐free workflow for electron treatments.

To enable a fair analysis, both the 3D camera and CT scanned structures were exported from the TPS, and converted into .stl files using the same ESAPI script, that is, both CT data and 3D camera data were analyzed using the exact same workflow and algorithms, such that any processing differences are negated. The two body surfaces were then compared using CloudCompare^10^ (https://www.danielgm.net/cc/). This equal processing of the CT‐scanned and 3D‐camera body surfaces equalizes any algorithm dependent effects such as mesh density, or cropped area when generating the .stl files. Specifically, when converting between .stl and DICOM formats, the CT‐slice resolution of the DICOM format is introduced. This can be seen by comparing Figs. 4(e) and 4(f), which are the same contour before and after converting to DICOM format. By exporting both.stl files from the TPS, they have gone through the same software processing.

Notably, the handheld 3D camera has sufficient spatial resolution to capture the phantom nose and ear contours. The largest deviations were seen around screws on the forehead that the 3D camera did not capture [red dot in Fig. 4(g)]. The handheld 3D camera system requires a person to be close to the patient. Although the scans in this work were performed using handheld cameras with the patient on the treatment table, the system was found to be convenient to use in a preparation or exam room, which may save linac or simulator time. The alternative setup of an OBI‐mounted Intel D415 3D camera system was found to provide an unobtrusive way to capture the patient surface. By mounting the Intel D415 camera above the kV detector panel of a radiotherapy linac, and connecting it to a gantry‐mounted laptop, a steady rotation of the camera view can be achieved, with minimal engineering [Fig. 1(a)]. This camera position may also be used to improve or extend the field of view for on‐treatment CBCT, as discussed in Jenkins et al^6^. The OBI‐mounted Intel camera was found to provide surface maps of equivalent accuracy to the handheld structure sensor, as might be expected from the similarity of the underlying technology. For detailed quantitative analysis of the performance of the Intel Real Sense 400‐series depth cameras we refer the reader to existing publications.^16^, ^17^, ^18^, ^19^

**Fig. 6:**
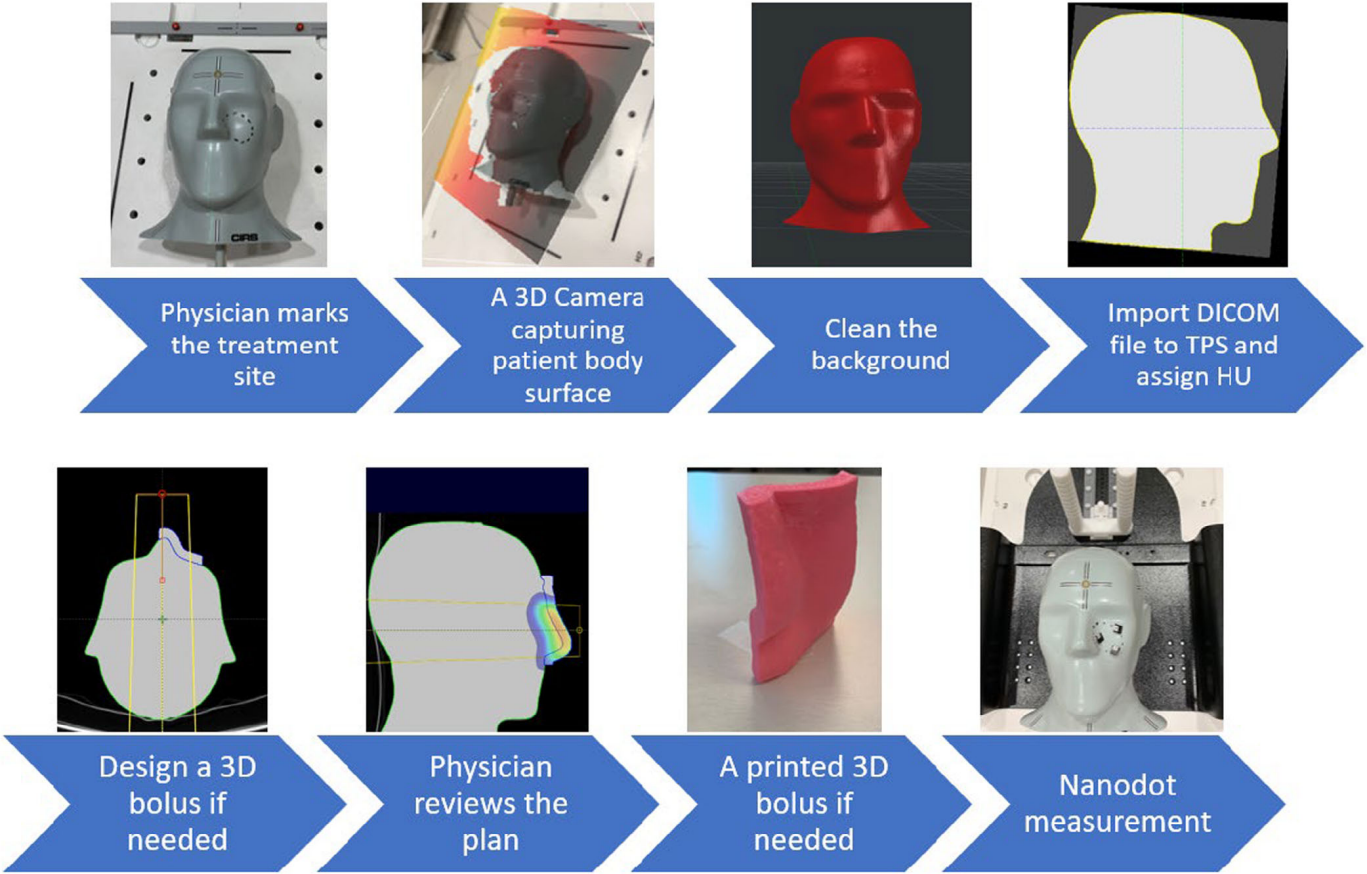
A workflow diagram for the CT‐less electron radiotherapy simulation.

Once dosimetric significance and handheld 3D camera spatial accuracy were both established, a clinical workflow was developed (Fig. 6). This workflow may also be adapted to use commercial surface imaging systems already designed for radiotherapy linac vaults, for example, Vision RT. However, they usually do not have a streamlined ability to export 3D captured surfaces. Furthermore, they are often very expensive and not financially affordable for some cancer centers. The single 3D‐camera workflows developed here has the advantage of low cost ($100‐$500) and minimal hardware installation requirements, making them more beneficial for resource‐limited settings.

Since the 3D camera can only capture the surface of the body, it cannot estimate how deep the lesion penetrates. If the depth of the disease to be treated is unknown, and there is no diagnostic imaging, a treatment planning CT scan is still required. Where a concern, setup error and its consequences on coverage can be investigated by calculating the plan with different shifts and different gantry angles. To ensure that day‐to‐day setup variation is minimized, the linac‐mounted MV or kV imaging systems may be used. A fixed surface imaging system such as C‐Rad, Vision RT, or HumediQ, may also aid the reproducibility of the setup. An additional device also means more QA, although the 3D camera is fairly stable. We recommend a scale check every month and after a software upgrade. The scale check is to make sure the units used in the software did not change. Since the exported .stl or .ply file is unitless, the software needs to assume some units, and thus if there is a unit change it will change the size of the scan.

### 4.1 Conclusion

Three‐dimensional cameras are a novel technique to capture irregular body surfaces and improve accuracy of electron dosimetry compared with traditional calculations. The 3D camera surface capture method avoids unnecessary patient exposure to ionizing radiation, and is easy to implement with low equipment cost (under $500) and short training times for staff (<2 hrs). The tools and workflow developed here are useful for electron radiotherapy planning of face and limb treatment sites, where the 3D camera captured surface provides an intermediate between a full CT scan and a simple flat‐phantom calculation. These same tools may also be used to create 3D printed patient specific devices such as bolus, skin collimators, and masks.

## Conflict of interest

The authors have no relevant conflicts of interest to disclose.

## Author contributions

Lawrie B. Skinner, Piotr Dubrowski, and Yi‐Chun Wang performed the experiment and analyzed the data. Rick Knopp and Alyssa Limmer collected the data. Karl Bush wrote the script to export the contour. Nicholas Trakul, Lynn Million, Carol Madellaine Marquez, and Amy S. Yu are the PIs of the project. They conceived and designed the analysis. All the authors wrote the paper.
